# Ki-67 is a prognostic marker for hormone receptor positive tumors

**DOI:** 10.1007/s12094-015-1472-y

**Published:** 2016-01-07

**Authors:** M. E. Pérez-López, J. García-Gómez, M. T. Alves, A. Paradela, J. García-Mata, T. García-Caballero

**Affiliations:** 1 Department of Oncology, University Hospital of Ourense, University of Vigo, C/Ramón Puga 52-54, 32005 Ourense, Spain; 2 Research Unit, University Hospital of Ourense, University of Vigo, Ourense, Spain; 3 Department of Pathology, University Hospital of Ourense, University of Vigo, Ourense, Spain; 4 Department of Morphological Sciences, School of Medicine-University Clinical Hospital, University of Santiago de Compostela, Ourense, Spain

**Keywords:** Ki-67, Tumor proliferation marker, Immunohistochemistry, Prognostic factor, Overall survival

## Abstract

**Purpose:**

To evaluate the utility of Ki67 as a prognostic marker in Luminal B node-negative breast cancer patients.

**Methods:**

We identified 888 patients with invasive breast carcinomas who underwent surgery between 1997 and 2004. Several classical factors were collected: age, tumor size, node involvement, tumor grade, estrogen and progesterone receptors, HER2 and Ki-67 expression. We analyzed if these parameters could be considered as a prognostic factor. In early Luminal B group, we investigated which of the following biological features provide information about bad prognosis: lack of progesterone receptor expression, HER2 overexpression/amplification or high Ki-67 value.

**Results:**

The majority of patients were alive and without relapse of tumor at the moment of the analysis (70 %). The prognostic factors founded in multivariate analysis were: tumor size, node involvement, grade 3 and Ki-67 expression. When we stratified the sample by immunohistochemistry (IHC) in tumor subtypes, we assessed 680 patients and we observed 191 Luminal B tumors. The biological parameter related to the worst survival in absence of nodal involvement was Ki-67 value.

**Conclusions:**

Ki-67 represents an additional predictor of survival in Luminal B node negative breast cancer. Conversely, neither Progesterone-receptor nor HER2 status proved prognostic significance in this group in our study.

## Introduction

Breast cancer is a heterogeneous disease with different biological and clinical behavior and prognosis [1, 2]. Saint Gallen Consensus established the classification of breast cancer subtypes by IHC as a surrogate of their genomic profile and suggested suitable treatment options in each one [3, 4].

Although classical prognostic factors keep their meaning [5], nowadays is common, in development countries, detecting early breast cancer because the screening programs. Therefore, it is necessary to seek others prognostic factors different from size, nodal involvement or tumor grade to adjust the complementary choice of treatment after surgery.

The significance of Ki-67 as a prognostic and predictive marker has been widely studied. Nevertheless, it has not still been considered as an independent prognostic factor [6]. In the genomic era, IHC classification of tumors is permitted and recognized for choosing the adjuvant treatment [3, 4]. The evaluation including estrogen receptor (ER) and progesterone receptor (PR) expression, HER2 expression and Ki-67 assessment has provided similar results than Oncotype in recurrence prediction with lower cost and more accessibility [7, 8]. It is an important point in Luminal B tumors without nodal involvement. Even though the literature has focused on the great value of hormonal receptors and HER2 expression, Ki-67 had shown to be of no less importance.

## Patients and methods

It is a descriptive study of 888 patients with invasive breast cancer without metastatic disease from the CHUO (Hospital of Ourense in Spain) Tumor Registry between 1997 and 2004. We analyzed traditional prognostic factors and their relation with breast cancer-specific survival.

We analyzed 680 samples by IHC, due to lack of data about some markers in the whole population. After the distinction between Luminal A and B tumor fenotypes through HER2 and Ki-67 expression [4, 9], a better classification has been proposed. We classified our patients in subtypes following the Saint Gallen criteria: Luminal A (ER+ and/or PR+, HER2− and Ki-67 <14), Luminal B (ER+ and/or PR+, HER2+ and/or Ki-67 ≥14), Erb-B2 (ER−, PR− and HER2+) and Triple Negative (ER−, PR− and HER2−) [3].

Even though the evaluation of markers is a review of pathological reports, a sole pathologist was responsible for the Ki-67 quantification and the paraffined samples underwent a local staining. So, the variability inter-observer was droved out. A substantial expertise in the assessment of Ki-67 with good intraobserver reproducibility in this condition is reported by Polley in an international Ki-67 reproducibility study.

This study was approved by the Ethics Committee of Research of Galician System Health-SERGAS, Spain.

More than a half of women were diagnosed in early stages of the disease. Half of our patients showed tumors smaller than 2 cm with negative nodes (pT1N0).

Patients received chemotherapy and radiation therapy in consonance to the regularly updated protocol of Oncology Medical Service of Hospital of Ourense, Spain. Chemotherapy schedules were based on anthracyclines, CMF and in a lesser extent, taxanes. Almost 60 % of patients received chemotherapy. Anthracyclines and CMF were the most used chemotherapy schedules, with a slight preponderance of the first. The combination anthracycline and taxane was only used in 8 % of patients. Hormonotherapy was based on tamoxifen and aromatase inhibitors. 80 % of women received hormonotherapy, mainly tamoxifen (almost 60 %). 5 % of the patients received Aromatase inhibitors monotherapy. Adjuvant Trastuzumab was not used in the period of our study, because it was not still approved in the adjuvant setting.

Patients were regularly followed-up until October 2010.

Expression of the estrogen and progesterone receptors was analyzed by IHC using monoclonal antibodies: clon 1D5 (Dako, Glostrup, Denmark) and clon 1A6 (Menarini, Novocastra, Italy), respectively and Envision (Dako) as detection system. According to the extension and intensity of the staining, tumors were classified as strong positive (+++), moderate (++), weak (+) and negative (−). Threshold level for positivity was established in 10 % of the cells showing nuclear positivity of any intensity.

Amplification or overexpression of HER2 was analyzed using HercepTest kit (Dako). Intense and complete staining of the membrane in more than 10 % of tumor cells was interpreted as positive (score 3+). Tumors with weak to moderate membrane expression in more than 10 % of the cells were interpreted as equivocal (2+). In these cases FISH analysis was performed and tumors with HER2 amplification were considered positive.

**Fig. 1:**
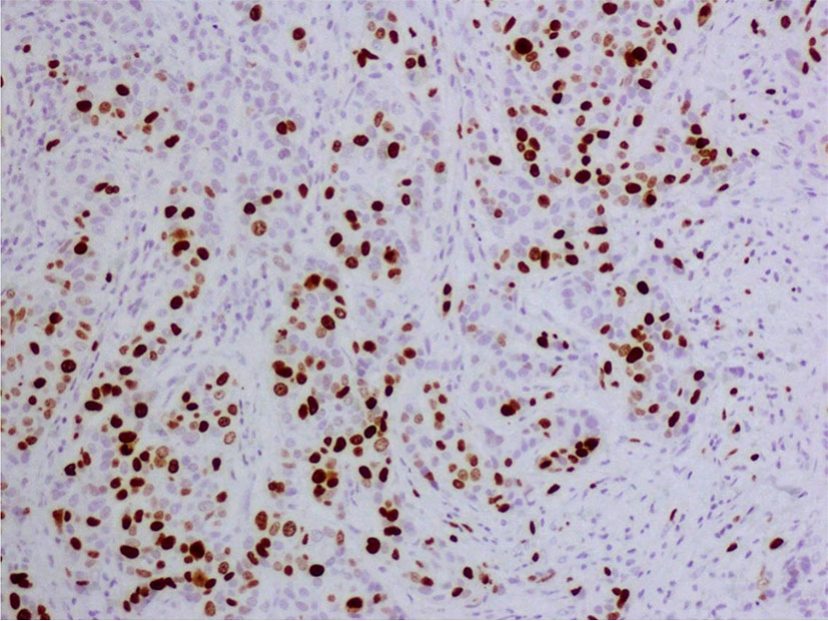
High expression of Ki-67 (>20 %)

**Fig. 2:**
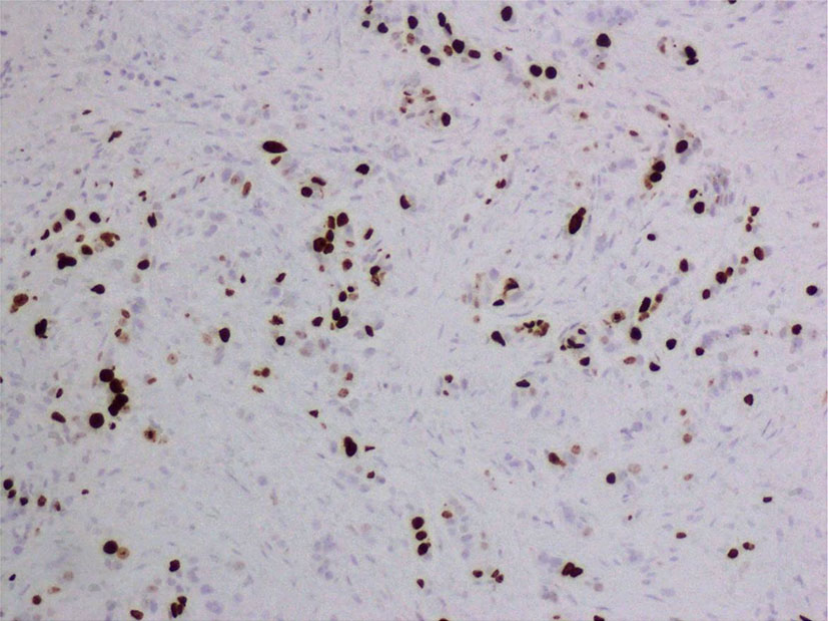
Medium expression of Ki-67 (10–20 %)

**Fig. 3:**
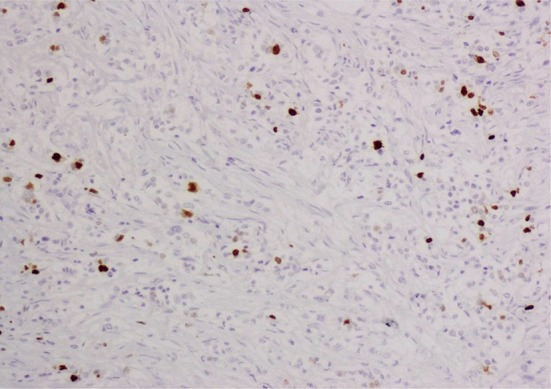
Low expression of Ki-67 (<10 %)

Regarding the Ki-67 measure, IHC staining was used by human Ki-67 monoclonal antibody (clon MIB-1, DAKO) and Envision as detection system. MIB-1 has been considered as a gold standard by the recommendations from the International Ki-67 Assessment in Breast Cancer Working Group [10]. The Ki-67 percentage score was defined as the percentage of positively stained malignant cells among the total number of malignant cells. In order to ensure the quality of the staining, positive control tissues are used (Figs. 1, 2, 3).

The cut-off point of 14 % was accorded regarding the current recommendations [3, 4, 9, 10]. The complete sample is studied and checked for immunostaining tumor cell nuclei. Score is determined considering the whole tumor section (not only the hot spots of the carcinoma or the most evident positive parts within the invasive segment).

### Statistics

The data were collected in a database designed for this purpose. The SPSS 17.0 program was used for the analysis.

Firstly, a descriptive analysis of the population was performed. The qualitative variables were described with absolute and relative frequencies, and the quantitative variables were described with central tendency measures: average, medium, style and dispersion standard deviation, range or trust interval to 95 %.

A bivariant analysis was performed with the statistic tests that correspond according to the type of variable: Chi square for qualitative variables and *t* of Student Fisher for quantitative variables. Non-parametric tests were applied when the variables did not follow a normal distribution.

Global survival and disease-free survival, using Cox regressions and Kaplan–Meier method, was estimated for the classic prognostic variables.

The results were considered significant when the obtained values were *p* < 0.05.

## Results

Regarding the patients’ conditions at the moment of the data collection (October-2010), 70 % of them were alive. There was a similar proportion between deceased patients due to breast cancer and deceased patients due non-tumor related causes (13 %). The aging of the sample justifies our findings and forces us to be prudent at the time of deciding and adjusting individually complementary therapy, especially in this group of women. Less than 20 % of the patients underwent a relapse, in a similar way to the Spanish average.

Age of patients ranged from 27 to 96 years, with a mean of 62.1 and a median of 62 years (Table 1). The 57 % of breast cancer patients were over 60 years. This is a foreseeable fact in an aged province like ours, which shows an inversion in its population pyramid. Only a 7 % of women were <40 years old. Age proved to be a survival prognostic factor in univariate analysis. However, age did not achieve prognostic significance in multivariate analysis.

**Table 1:** Baseline characteristics of patients

Patient characteristic	Subjects *N* (%)
Age	62.10 ± 14.60 [27–96]
Tumor size	
T1	441 (49.7 %)
T2	336 (37.8 %)
T3	60 (6.7 %)
T4	42 (4.8 %)
Not classified	9 (1 %)
Lymph node status	
N0	459 (51.7 %)
N1	229 (25.8 %)
N2 and N3	147 (16.5 %)
Not classified	53 (6 %)
Tumor grade	
G1	208 (23.5 %)
G2	343 (38.6 %)
G3	232 (26.1 %)
Not classified	105 (11.8 %)
Histology type	
Ductal	743 (83 %)
Lobular	67 (7.5 %)
Tubular	22 (2.5 %)
Mucinous	19 (2.1 %)
Medular	16 (1.8 %)
Papilar	13 (1.7 %)
Other	8 (0.9 %)
p53 mutation	
Yes	544 (62 %)
Not	109 (13 %)
Not classified	235 (25 %)
Ki67	
<14	410 (47 %)
≥14	244 (28 %)
Not classified	234 (25 %)
Estrogen receptor	
Positive	666 (75 %)
Negative	178 (20 %)
Not classified	44 (5 %)
Progesterone receptor	
Positive	456 (51.5 %)
Negative	381 (43 %)
Not classified	51 (5.5 %)
Her2	
Positive	596 (68 %)
Negative	92 (10.5 %)
Not classified	200 (21.5 %)
IHC subtypes	680 (77 %)
Luminal A	360 (40 %)
Luminal B	194 (22 %)
ErbB2	26 (3 %)
Triple negative	100 (12 %)
Not classified	208 (23 %)

More than a half of women were diagnosed in early stages of the disease. Half of patients had tumors smaller than 2 cm without node involvement (pT1N0). Therefore we must seek other new prognostic factors that help us to decide the subsequent treatment of each patient of this group.

The most common histological grade was G2. The G3 (undifferentiated) tumors represent 30 % of the cases and it was the only one that proved prognostic value in all statistical analysis performed.

The expression of estrogen receptors was observed in three-fourth of the study population and the progesterone in a half. Both of them have prognostic value only in univariate analysis.

The HER 2 amplification was identified in 10 % of the women. It kept prognostic value in univariate analysis. The period of inclusion (until 2004) was chosen in order to evaluate prognostic factors before the start of trastuzumab therapy as an adjuvant treatment in patients with overexpression HER2. Thus, we can obtain a wider approximation to the intrinsic biological behavior of this women subgroup.

The mutation of p53 was verified as a survival prognostic value in univariate analysis. In multivariate analysis, p53 mutation and HER2, estrogen and progesterone expression lost their prognostic value.

Nearly half of the patients showed low levels of Ki-67 (<14 %). These data were collected as a numerical value in the pathological report. It kept prognostic value in univariate analysis as well as in multivariate.

In our study tumor size, nodal involvement, histologic grade III, and high Ki-67 score constitute independent prognosis factors (Table 2). The specific survival of breast cancer is determined by these parameters as has been reported previously in the literature [5]. High Ki-67 score (≥14 %) increased 2.73 times the mortality risk of breast cancer (*p* < 0.01).

**Table 2:** Prognostic factors: multivariate analysis

Variable	*p*	Exp (B)	95 % CI Exp (B)
Age	0.79		
40–60 years	–	–	–
<40 years	0.55	1.27	(0.58–2.79)
>60 years	0.60	1.16	(0.66–2.05)
Tumor size	0.01		
T1	–	–	–
T2	0.03	2.23	(1.09–4.58)
T3	0.01	3.60	(1.34–9.65)
T4	<0.01	7.56	(2.89–19.78)
Nodal involvement	<0.01		
N0	–	–	–
N1	0.05	2.01	(0.99–4.10)
N2–N3	<0.01	4.11	(1.98–8.53)
Tumor grade	0.02		
G1	–	–	–
G2	0.09	3.62	(0.82–17.97)
G3	0.01	6.36	(1.43–28.27)
Ki67 expression	<0.01		
<14	–	–	–
≥14	<0.01	2.73	(1.35–5.38)
p53 mutation	0.97		
Negative	–	–	–
Positive	0.97	1.01	(0.55–1.87)
ER expression	0.77		
Negative	–	–	–
Positive	0.77	1.11	(0.56–2.20)
RP expression	0.07		
Negative	–	–	–
Positive	0.07	0.58	(0.32–1.06)
Her2 overexpression	0.29		
Negative	–	–	–
Positive	0.29	1.31	(0.76–2.51)

When the patients were stratified in tumor subtypes by IHC methods as previously defined, we observed that 194 belonged to luminal B subtype. 161 of them (83 %) showed a high Ki-67 and 30 (15.5 %) a low Ki-67; 3 patients (1.5 %) had unknown value.

When we analyzed node status in Luminal B subgroup we found 87 patients without node involvement (N0). This was the tumor subtype that, in absence of node involvement, showed a worse prognosis (even worse than the HER2 subtype-where there were only five patients and none relapsed- and triple negative) (Table 3).

**Table 3:** Recurrence subtypes

	Luminal A	Luminal B	ErbB2	Triple negative	*p*
	*N* (%)	*N* (%)	*N* (%)	*N* (%)	
Negative nodes					0.004
No recurrence	193 (95.5)	73 (83.90)	5 (100)	44 (84.60)	
Recurrence	9 (4.5)	14 (16.10)	0	8 (15.40)	
Total	202 (100)	87 (100)	5 (100)	52 (100)	
Positive nodes					0.001
No recurrence	112 (81.20)	70 (68.60)	9 (47.40)	23 (52.30)	
Recurrence	26 (18.80)	32 (31.40)	10 (52.60)	21 (47.70)	
Total	138 (100)	102 (100)	19 (100)	44 (100)	

**Fig. 4:**
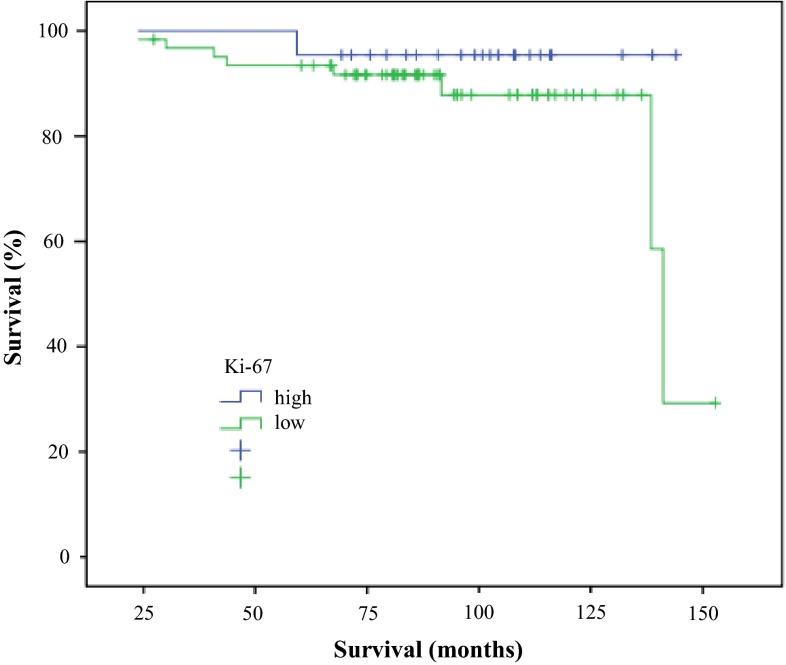
Survival in luminal B subtype and Ki-67 value

In this subgroup 26 % of patients showed low Ki-67 and 74 % high Ki-67, a 5 % of patients with low Ki-67 and a 13 % of patients with high Ki-67 died (Fig. 4). Studying relapses in the same subgroup, and their relation with HER2, PR and Ki-67 expression, we observed a 20 % relapse rate in patients with PR expression and a 10 % in absence of PR expression; a 20 % rate of relapse HER2 negative women and a 12 % rate in HER 2 positive. The rate of relapse in patients with low Ki-67 was a 9 % and a 18 % when Ki-67 was ≥14 %.

## Discussion

The biological and prognostic heterogeneity of breast cancer is widely recognized [1, 2]. This diversity determines a great deal of the risk of relapse of each tumor, and therefore, the different election of complementary treatment, especially in early stages [3, 4].

Right now, to the prognostic value of the “burden tumor”, meaning the size of the tumor and the node involvement, should be added other tumor biological factors. As in other published studies, in our population, tumor size, as well as node involvement turned out to be survival prognostic factors, in univariate analysis, as well as in multivariate analysis, maintaining their independent prognostic value [5]. In contrast, ER and PR expression and HER2 overexpression lost their independent prognostic value in multivariate analysis. Nevertheless, one of the limitations of our study in this assessment is the small percentage of patients with known HER2 overexpression.

In the same way, the prognostic value of tumor grade has been highlighted [5]. It maintained significance in uni- and multivariate analyses, but exclusively in tumors undifferentiated (G3), increasing 6.36 times the risk of death by breast cancer (*p* = 0.02). Other studies have not given this prognostic quality to tumor grade either [11], recommending the routine study of the Ki-67 antigen expression and giving more value to this one. Despite, other studies have limited this worthiness to women with hormone-sensitive tumors which are moderately differentiated [12]. A recent publication has reported a correlation between higher Ki-67 value and Erb-B2, triple negative and luminal B-HER2 negative tumors. Besides histological grade was not informative for estimating proliferation in luminal B HER2 negative, being necessary other tools like Ki-67 index [13].

We also observed that Ki-67 was a factor related with survival in multivariate analysis. Several meta-analyses demonstrated the prognostic influence of this factor [6, 7, 11, 14–16]. Two of them [14, 15], which included 12,155 and 32,825 patients, respectively, led to the same conclusion even when different inclusion criteria, different Ki-67 assessment methods and different cut-points were used. In spite of these differences, the results on the prognostic value of Ki-67 were consistent.

In search of elements that can help us to identify those women with a favorable prognosis in order to avoid overtreatment, we have continued to study this marker [6, 16, 17]. Using samples of randomized studies, where a second Ki-67 centralized analysis was performed, Luporsi recently recognized Ki-67 as an independent prognostic factor of free relapse survival. It reached a level of evidence IB [16]. It did not achieve the IA level because none of the revised studies had been specifically designed to be evaluated it as a prognostic factor.

A more recent meta-analysis about the prognostic significance of Ki-67 recognizes that the debate is still open, even when most of the studies have established their relation with the free relapse survival and the specific breast cancer survival. However, the biggest inconvenient to establish a standard has been its measurement [6, 7]. The published recommendations about its determination will help this purpose [10].

When we divided our series of 680 patients in tumor subtypes by IHC, according to the Saint Gallen criteria, [3] and we studied the prognosis of them, with and without node involvement, we observed that luminal B N0 patients had the worse prognosis. We tried to identify which of the variables that categorized them as Luminal B, the absence of PR expression, the HER 2 overexpression or Ki-67 ≥14 levels were the ones that conferred this worse prognosis.

Of the 87 patients with luminal B tumors, those with PR expression, absence of HER 2 overexpression and high levels of Ki-67, underwent the majority of relapses. Even though, there is not the unique research with similar outcome regarding HER2 overexpression [18]. From our point of view, this fact underlines the value of Ki-67 even more.

As early as 2005, Urruticoechea reviewed 40 studies that included more than 11,000 patients and he found that Ki-67 was a prognostic factor in N0 patients in the uni- and multivariate analysis [19]. A later report, which studied breast cancer prognostic factors after 5 and 10 years in patients with pT1N0M0 tumors, demonstrated that Ki-67 had a prognostic value and that it was the only factor that did not lose this value over time [20].

In the genomic era, because of the cost and availability of gene microarrays, IHC classification of the tumors represents a surrogated marker of gene profiles in the daily routine and determination of Ki-67 takes on a great significance [3, 4].

Ki-67 is the most important cell proliferation marker in Oncotype, which constitutes the biggest determinant in the gene expression profiles [21, 22]. These genomic profiles are applied to predict the risk of relapse, especially in hormone-sensitive patients. However, comparing two of the most accepted, PAM 50 and Oncotype, they had differences in the risk classification of the patients [23]. There was no display of benefit in the prediction of the relapse risk by Oncotype face to face an IHC profile, IHC4, consisting in ER, PR, HER 2 and Ki-67 expression and supposing a great accessibility in the daily routine, besides supposing IHC a great economical saving [8]. The lack of a greater exactitude in the relapse prediction in Oncotype, before IHC in which Ki-67 is included, has also been reflected in other studies [24, 25].

Although some reports have manifested the predictive value on Ki-67 [26–29], there is no agreement on the matter [16, 30, 31], even when its association with complete pathological responses in neoadjuvant treatment with evidence level IIA is recognized [16].

In summary, on the current rise of the gene expression profile studies and their use in the calculation of relapse risk in breast cancer [32], we must not forget that the classification of tumors based in TNM system and IHC including Ki-67 assessment has not lost its prognostic value, keeping greater efficiency and accessibility, and being able to be used with security and lower cost in the clinical routine. Classical factors as size of tumor, nodal involvement and tumor grade sustain their prognostic role. However, it should be completed now with new biological tumor parameters. Ki-67 has been demonstrated as an independent and additional predictor of survival in multivariable analysis. In the genomic era Luminal B node negative breast cancer by IHC has proved being a prognostic factor. Conversely, neither progesterone-receptor expression nor HER2 status has shown it in this group. Standardizing Ki67 right assessment will improve estimation of breast cancer recurrence risk.
